# Autofluorescent Activity of Thermosensitive, Hemostatic, and Wound Healing Biopolymer Hydrogels

**DOI:** 10.3390/gels11040301

**Published:** 2025-04-19

**Authors:** Sergey I. Petrushenko, Mateusz Fijalkowski, Kinga Adach, Vladimir Lebedev, Katerina Lebedeva, Anna Cherkashina, Kateryna I. Rudnieva, Natalja P. Klochko

**Affiliations:** 1 Institute for Nanomaterials, Advanced Technologies and Innovation, Technical University of Liberec, 46117 Liberec, Czech Republic; kinga.adach@tul.cz; 2 School of Physics, V.N. Karazin Kharkiv National University, 61022 Kharkiv, Ukraine; 3 Department of Plastics and Biologically Active Polymers Technology, National Technical University “Kharkiv Polytechnic Institute”, 61002 Kharkiv, Ukraine; vladimirlebedev1980@ukr.net (V.L.); oazis.ruk@gmail.com (K.L.); annikcherkashina@gmail.com (A.C.); 4 Physical and Chemical Research Department, National Scientific Center, Hon. Prof. M. S. Bokarius Forensic Science Institute, 61177 Kharkiv, Ukraine; rudneva770@ukr.net; 5 Department of Micro- and Nanoelectronics, National Technical University “Kharkiv Polytechnic Institute”, 61002 Kharkiv, Ukraine; klochko.np16@gmail.com

**Keywords:** biopolymer, thermosensitive hydrogel, fluorescence, gelatin, alginate, humic acids, aminocaproic acid, delivery of hemostatic drugs

## Abstract

Thermosensitive biopolymer gelatin–alginate hydrogels are promising for use as dressings for wound healing and drug delivery. This work presents fluorescence arising from the internal fluorophores of alginate and gelatin biopolymers in thermosensitive hydrogels modified with calcium- and sodium-containing humic acids before and after their impregnation with the hemostatic drug aminocaproic acid. A new approach of using fluorescence emission spectra, along with the analysis of morphological features, optical properties, and the elemental composition of dried hydrogels, is used as a tool for monitoring the ability of these hydrogels for the thermosensitive delivery of a hemostatic drug. A comparative analysis made it possible to select the optimal composition of hydrogels suitable for the targeted delivery of aminocaproic acid through a gel–sol transition at physiological temperatures. Optimal concentrations of sodium-containing humic acids in gelatin–alginate hydrogels of 2.5 wt.% and 5 wt.% provided a gel–sol transition temperature of about 37 °C. The quantum yield of fluorescence of 8–10% upon introduction of 20 wt.% aminocaproic acid into these hydrogels indicates that this hemostatic drug does not destroy three-dimensional networks formed by molecules of gelatin, alginate, and humic acids, the gel–sol transition temperature for which is maintained at a physiological level without significant contracture of the wound dressing.

## 1. Introduction

Sustainable natural bio-origin materials, particularly the hydrophilic biopolymer protein gelatin (Gn) extracted from animal and fish waste and the anionic polysaccharide sodium alginate (SA) derived from brown algae, are promising building blocks for the creation of next-generation flexible and soft electronics due to their excellent biocompatibility, biodegradability, and multiple active sites [1,2]. The morphological diversity of biopolymers and their composites, as well as their numerous functional groups for modifications, make them attractive for the creation of actuators and sensor devices, including for temperature-controlled drug delivery [1,2,3,4]. The authors [5,6,7] presented biocompatible, biodegradable hydrogel smart materials that were used in the design of soft robots and sensors, for which biopolymer hydrogels acted as active primary materials. The authors [8] demonstrated the stimulus–response transformation from strong to flexible in response to water stimuli in a dried hydrogel made of gelatin crosslinked with melamine–formaldehyde resin. The reversible solvation-controlled elasticity in the smart gel [8] was demonstrated by the fact that when exposed to water, the tough dry gel can reversibly transform into an elastic wet gel by absorbing water. Recently [9], a natural biopolymer hydrogel based on a gelatin–graphene nanocomposite was used in a sensor operating in the temperature range from −13 °C to 37 °C to detect ice formation at sub-zero temperatures, recording temperature-dependent open-circuit voltage. When the number of hydrogen bonds inside the biopolymer changes with temperature, the accumulation of charge carriers at the boundary of Gn with graphene nanoparticles and with metal electrodes changes, demonstrating the output electrical signal of the temperature sensor [9]. In [10], the temperature reversibility of hydrogen bonds in a biocompatible hydrogel based on polyvinyl alcohol, phytic acid, and gelatin was used as a temperature-sensitive adhesive for flexible electronic sensors designed to record electrophysiological signals in human health monitoring. This hydrogel with temperature-controlled mechanics can firmly adhere to the skin and detect electrophysiological signals under hot compression and, at the same time, be easily removed under cold compression [10]. The sol–gel transition is a popular detection strategy [6,11] that uses external stimuli, such as small changes in temperature, to trigger the breakdown or construction of hydrogel scaffolds. When heated to a certain temperature, the structure of the gel changes and turns into a sol, which affects the signaling molecules embedded in the hydrogel scaffolds by, for example, releasing them from the hydrogel, thereby generating detectable signals. The above sol–gel transition and thermosensitive swelling/de-swelling function are currently used in gelatin-based drug delivery systems [6,12,13]. These systems provide temperature-responsive delivery of the therapeutic agent, as well as its release and subsequent transport through the target site. However, the problem with using gelatin hydrogel wound dressings is the low melting point of gelatin, which is in the range of 28–31 °C for mammalian gelatin and in the range of 11–28 °C for fish gelatin [12]. Thus, to deliver drugs from a wound dressing, the Gn polymer chains must be crosslinked to create a double network of nanocomposite hydrogels that is mechanically stronger and has a melting point near the physiological temperature of 37 °C. In particular, sodium alginate is not only able to treat inflammatory diseases [13] and is suitable for temperature sensors as a polyelectrolyte hydrogel with ionic conductivity increasing with temperature [14,15,16], but it can also improve the mechanical strength of hydrogels when dispersed in a hydrogel system [15]. Due to its amphiphilic properties, SA has also been demonstrated to be a good dispersant [16], making alginate-based hydrogels very promising biocompatible and biodegradable candidates for use as drug delivery systems [6]. According to [17], by modifying and combining different gelling agents and crosslinkers, stimuli-responsive hydrogels can be imbued with various properties, such as controlled gelation, degradation, or changes in stiffness. Typically, induced crosslinking between gelling agents results in an increase in stiffness or, at some point, gelation [17]. The above explains the relevance of research on gelatin–alginate hydrogels with controlled sol–gel transitions used for the delivery of hemostatic drugs. Moreover, the combination of various crosslinks can be another way to create functional hydrogels with high swelling capacities and mechanical tunability, since hydrogel networks can absorb a large amount of biological fluids [17], which further makes gelatin–alginate hydrogels promising materials for hemostasis.

Recently, polymer hydrogels have been found to respond to exogenous stimuli such as light by exhibiting intrinsic fluorescence [18,19,20,21], which is the process of light emission by a material that absorbs light at a lower wavelength (higher energy) and emits light at a longer wavelength (lower energy). Intrinsic fluorophores include naturally occurring materials such as green fluorescent proteins [18], including gelatin [20], and the polysaccharide sodium alginate [21,22]. According to [18,19], the fluorescence of hydrogels without adding fluorescent substances to them from outside through chemical bonding or physical doping, i.e., the autofluorescence associated with the typical fluorophore groups of hydrogels, exists everywhere in carbonyl-containing gels. In polymer autofluorescent hydrogels, the fluorescence intensity increases with decreasing water content, since the lower the water content, the more restricted the movement of molecular chains. Thus, in a water-deficient environment, the ratio of electrons that return to the ground state from the excited state via non-radiative transitions decreases, which leads to a greater realization of such transitions in radiation mode, i.e., the aggregation-induced light emission described in [19]. The authors [19] suggested that crosslinked polymer chains in composite hydrogels may contribute to the reduction of non-radiative transitions and thus may further increase the fluorescence intensity due to additional bonds between different components of hydrogels, with subsequent increases in the rigidity of polymer chains. In some works [21,22,23,24,25], it was also noted that the greater the amount of the crosslinking agent, the higher the fluorescence intensity, and that the rigidity of molecular chains is effective in increasing the efficiency of fluorescence, i.e., the crosslinking structure favors the efficiency of fluorescence. At the same time, fluorescence is quenched by adding some specific metal ions to hydrogels [18,19,23,24,25]. Thus, fluorescence is successfully used for the self-monitoring of the gelation process since the fluorescence signal is closely related to the change in the internal structure of gels. In particular, according to [18,19,20,21,22], autofluorescence provides a reliable basis for detecting the gel structure and opens up new prospects for the application of hydrogels.

Recently, humic acids as a class of multifunctional macromolecular compounds with quinone, phenolic, carboxyl, and hydroxyl functional groups, which are obtained from humic substances in soil, brown coal, and peat by the oxidative decomposition of biomass [26,27], have been used as physical agents to improve the mechanical and thermal properties of gelatin hydrogels. According to [27], humic acids have an uncertain composition, which varies depending on their origin and production process. Commercial humic acids are extracted from peat and coal. Their chemical composition may vary depending on their geographical origin, age, climate, and biological conditions, which makes it difficult to accurately characterize these substances. The average elemental composition of humic acids extracted from different sources, including commercial ones, is approximately 50 at. % C, 35 at. % O, and 5 at. % H, with the remaining percentage distributed between N and S [27]. Humic acids are soluble in alkaline media, partially soluble in water, and, due to their amphiphilic nature, form micelle-like structures in neutral and acidic media [27]. In [26], humic acid from Sigma-Aldrich was chosen as a model moiety, although in fact it is partly sodium salts of humic acids (HANA). Since humic acids contain many functional groups (such as hydroxyl, ethoxyl, and carboxyl) and binding sites, they can react with calcium ions (Ca^2+^) to form partial calcium salts of humic acids (HACA), which may cause coagulation [28,29]. Due to their antioxidant, fungicidal/bactericidal, and anti-inflammatory activities, humic acids are useful in medicine and pharmaceuticals for wound healing [26,27].

In recent works [30,31,32], thermo-responsive hydrogels based on gelatin and sodium alginate were modified with the humic acids HANA and HACA for their delivery for wound healing purposes. In these works [30,31], the rheological properties, swelling, and compression of a hydrogel containing 6.4 wt.% SA and 14 wt.% Gn, as well as gelatin–alginate hydrogels with the addition of 2.5–7.5 wt.% HANA or HACA humic acids, were experimentally studied. Their semicrystalline structure, as well as non-covalent intramolecular bonds and intermolecular interactions, were investigated by X-ray diffractometry and Fourier-transform infrared spectroscopy. The obtained data were used to manufacture thermosensitive biopolymer hydrogel systems of gelatin–alginate–humic acid with a physiological melting temperature of ~37 °C, which is necessary for the successful delivery of their medicinal components to the wound for the purpose of its healing. Varying the concentration and type of humic acids in these hydrogels [30,31] allowed the authors to regulate the gel’s softening time on the human body in the range of 6 to 20 min, which provided the possibility of controlled prolonged delivery of components to wounds. Based on the study of the effect of Ca^2+^ ions on the properties of humic acids and ion exchange, as well as the interaction of humic acids, SA, and Gn with the formation of denser gel networks, approaches to regulating the rate of softening of hydrogels at physiological temperatures and their swelling, simulating the absorption of wound exudate, were proposed and implemented [30]. In addition, low shrinkage of the hydrogel surface due to the crosslinking of gelatin–alginate networks with humic acids has been experimentally confirmed [30], which is important for avoiding problems of wound contracture and contour deformations when using hydrogel dressings for wound healing. Loading gelatin–alginate–humic acid hydrogels with the hemostatic drug aminocaproic acid (AA) at a dose of ~0.2 g/mL resulted [31,32] in an almost threefold increase in their swelling, which facilitated the dissolution of AA in the hydrogel for its subsequent delivery to the wound. Experiments modeling the transmembrane transport of aminocaproic acid from the developed hydrogels confirmed their ability to quickly deliver up to 494 mg of AA from 5 mL of hydrogel at physiological temperature in less than 30 s to deep and hidden areas of hemorrhage, which reduces the blood clotting time to only 95 s, ensuring rapid hemostasis [32].

According to [33,34,35], humic acids contain their own fluorophores. The authors of [33] described the autofluorescence emission spectra of various humic acids at wavelengths (λ) in the range of 412–490 nm. In [34], fluorescence emissions at λ 330–530 nm were used as fingerprints for humic acids of different origins. In [35], humic acids from lake sediments were characterized by broad autofluorescence emission spectra in the range of 300–500 nm. Note that the position of the autofluorescence emission peaks in SA is 480 nm [21], while in Gn, the maximum autofluorescence emission peak was observed at λ 520 nm [20]. According to [36], dry gelatin powder was highly fluorescent, with an emission peak at λ 560 nm, and after swelling, the emission peak of Gn was at λ 460 nm.

This paper presents data on the micromorphology, elemental composition, and intrinsic fluorescence of thermosensitive gelatin–alginate hydrogels modified with the humic acids HANA and HACA before and after their impregnation with the hemostatic drug aminocaproic acid. Their analysis allowed us to explain the features of thermal sensitivity, swelling during the absorption of water and biological fluids, and compression and shrinkage during the drying of biopolymer gelatin–alginate–humic acid hydrogels with different compositions. The obtained data are used to clarify the mechanism of targeted drug delivery for successful hemostasis and subsequent wound healing, carried out by hydrogels with optimal compositions suitable for the delivery of hemostatic drugs through a gel–sol transition at physiological temperature, with rapid and abundant transmembrane transport to the wound site.

## 2. Results and Discussion

Table 1 presents the compositions of the hydrogels studied in this work.

**Figure 1:**
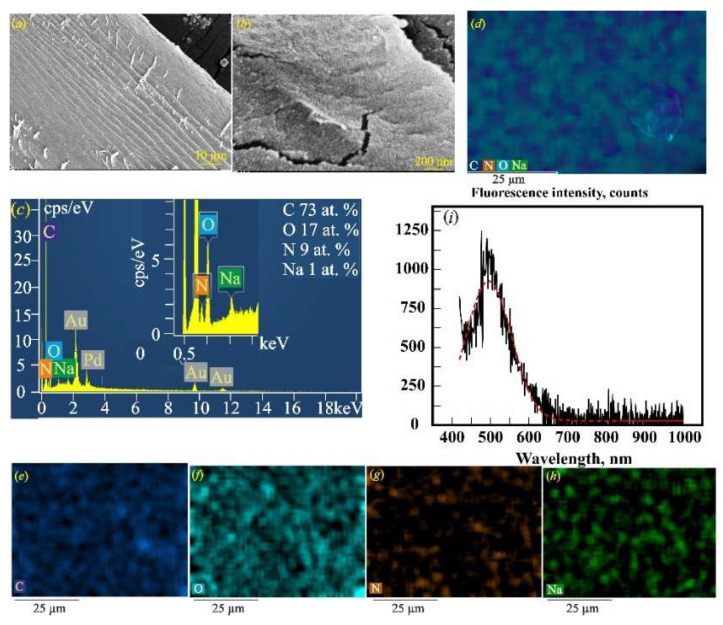
SEM images of the dried Gn_SA hydrogel with low (**a**) and high (**b**) magnification. (**c**) EDS spectrum of the dried Gn-SA hydrogel (the hydrogel is coated with a 50 nm thick Au80Pd20 film). (**d**) Overall energy dispersive X-ray spectroscopy (EDS) map of the dried Gn-SA hydrogel. Elemental EDS mapping of individual elements in the dried Gn_SA hydrogel: (**e**) C; (**f**) O; (**g**) N; (**h**) Na. (**i**) Fluorescence emission spectrum of the dried Gn_SA hydrogel at an excitation wavelength of 405 nm.

**Figure 2:**
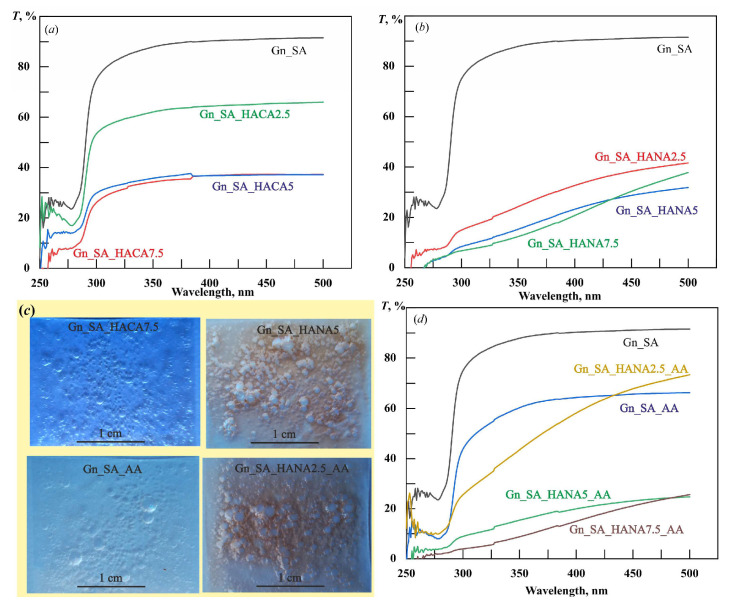
Optical transmission spectra (**a**,**b**,**d**) and photographs (**c**) of dried biopolymer hydrogel films (~0.5 mm thick) applied to glass substrates (measurements were made relative to bare glass substrates. The blue color of the photographs corresponds to the color of the table on which the samples were placed).

Figure 1a,b present the morphological characterization of dried gelatin–alginate hydrogels by scanning electron microscopy. The SEM image in Figure 1a shows the layered structure of Gn_SA. The cracks seen in Figure 1b are the result of the strong compression of the Gn_SA washer during its natural drying process. The elemental composition of Gn_SA, shown in the EDS spectrum in Figure 1c, contains elements inherent in biopolymers: carbon and oxygen from Gn and SA, nitrogen from Gn, and sodium from SA. The overall EDS map in Figure 1d and the corresponding elemental maps in Figure 1e–h show a relatively uniform distribution of C, O, N, and Na throughout the sample. The defect seen in Figure 1d may be due to the insufficient strength of the Gn_SA hydrogel and its large shrinkage due to compression during the sol/gel transition and subsequent drying, which, according to [30,37], may cause undesirable wound contracture and contour deformation when the hydrogel dressing is used for wound healing. Figure 1i shows the fluorescence emission spectrum of the dried Gn_SA hydrogel, with one broad peak with a fluorescence intensity of about 1120 counts. Its approximation in Figure 1i and the fit using the Gaussian function presented in Table 2 show that the peak’s center (*Xc*) is at a wavelength of ~500 nm and it has a full width at half maximum (FWHM) of 124 nm. The fluorescence quantum yield of the dried Gn_SA hydrogel is about 35%. According to the literature [20,21,36], this peak corresponds to the intrinsic fluorophores of the crosslinked Gn and SA biopolymers in the dried Gn-SA hydrogel. In [20], the major peak of gelatin at 520 nm was mainly due to the emission of flavin adenine dinucleotide (flavoprotein), and the minor emission peaks at 420 nm and 450 nm were attributed to collagen and nicotinamide adenine dinucleotide, respectively. Another three emission peaks of gelatin in [20], located at 585 nm, 635 nm, and 670 nm, were attributed to porphyrin compounds. Thus, the fluorescence observed in Figure 1i should be the combined emission band of the dominant fluorophores in the alginate anion, flavoprotein, collagen, and nicotinamide adenine dinucleotide of gelatin. In addition, Table 2 shows data on the melting temperatures *(T_GS_*) of hydrogels, which we obtained earlier in our works [30,32] based on rheological studies. The hydrogel Gn_SA has a *T_GS_* = 36.4 ± 0.4 °C, which corresponds to its relatively low strength. Figure 2 shows that the dried Gn_SA hydrogel film with a thickness of ~0.5 mm is transparent to UV–visible light with a transmittance *T* of up to 90% but absorbs ultraviolet light with a λ less than 290 nm. It should be noted that all dried hydrogels obtained in this work have high porosity; a large number of air bubbles are visible in the photographs of four hydrogel films in Figure 2c.

**Figure 3:**
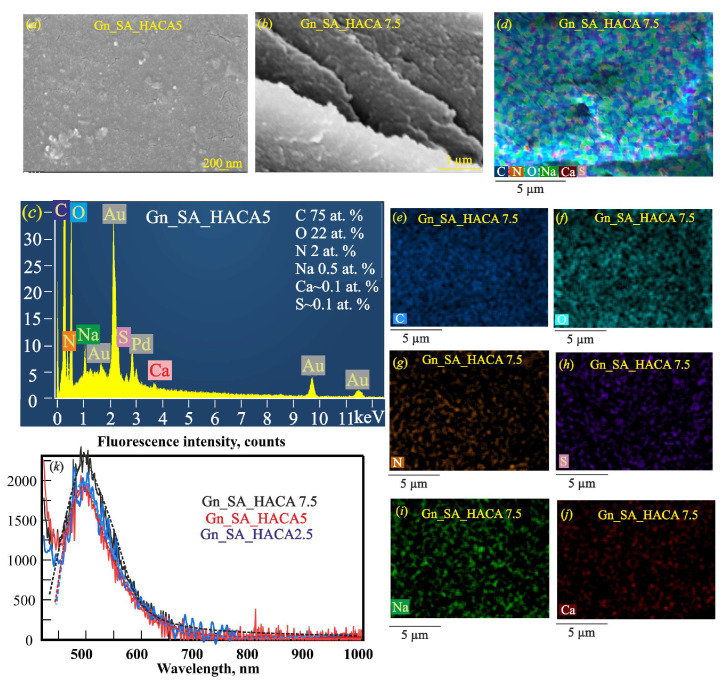
SEM images of dried gelatin–alginate hydrogels modified with HACA humic acids: (**a**) top view and (**b**) cross-section. (**c**) EDS spectrum of the dried Gn_SA_HACA5 hydrogel. (**d**) Overall EDS map of the dried Gn_SA_HACA7.5 hydrogel. Elemental EDS mapping of individual elements in the dried Gn_SA_HACA7.5 hydrogel: (**e**) C; (**f**) O; (**g**) N; (**h**) S; (**i**) Na; (**j**) Ca (all hydrogels are coated with 50 nm thick Au80Pd20 films). (**k**) Fluorescence emission spectra of dried gelatin–alginate hydrogels modified with HACA humic acids at an excitation wavelength of 405 nm.

Modification of gelatin–alginate hydrogels with HACA containing calcium ions increased the strength of the obtained Gn_SA_HACA2.5, Gn_SA_HACA5, and Gn_SA_HACA7.5 hydrogels, as described in [30], due to intermolecular bonds and especially ion exchange in the corresponding sols between Na^+^ from sodium alginate and Ca^2+^ from HACA, the Ca(OH)_2_ solution in which these humic acids are dissolved, and the subsequent ionic crosslinking of these hydrogels. According to [28,37], chain entanglement in such gelatin–alginate hydrogels containing calcium ions is the cause of gel contraction. This is shown in the SEM image in Figure 3a in the form of multiple fine cracks, which confirm the intense contraction of HACA-containing hydrogels. The SEM image of the cross-section of the dried Gn_SA_HACA7.5 hydrogel in Figure 3b shows its layered morphology. The EDS spectrum of the dried Gn_SA_HACA5 hydrogel in Figure 3c contains signals from C, O, N, S, Na, and Ca atoms. Among them, calcium and sulfur belong to HACA humic acids, which were obtained from sulfur-rich brown coal and thus are sulfur-containing humic acids similar to those described in [27,38]. The overall EDS map of the dried Gn_SA_HACA7.5 hydrogel in Figure 3d has many cracks that formed during the drying of the hydrogel; however, it shows a relatively uniform distribution of all of the above atoms throughout the sample, which is also confirmed by the EDS elemental maps in Figure 3e–j. The good solubility of high concentrations of HACA in gelatin–alginate hydrogels is confirmed by their relatively high transmittances in Figure 2a. However, with an increase in the content of HACA in hydrogels, the absorption of UV–visible light increased, which is explained, according to data [28,29], by the aggregation of humic acids with calcium ions. The UV–visible transmittance of the dried Gn-SA-HACA2.5 hydrogel film with a thickness of ~0.5 mm is up to ~62%. The UV–visible transmittance of the Gn_SA_HACA5 and Gn_SA_HACA7.5 films is up to ~33%. However, as can be seen in Figure 2, UV light with a λ less than 290 nm was strongly absorbed by all dried hydrogels.

The strengthening of the hydrogels in the series Gn_SA_HACA2.5, Gn_SA_HACA5, and Gn_SA_HACA7.5 is demonstrated according to [19,21,22,25] by the increase in their fluorescence emission intensity, as shown in Figure 3k and Table 2, to ~1900, ~1800, and ~2300 counts, respectively. Moreover, the peak position of the fluorescence emission is blue-shifted to *Xc* = 496 nm in Gn_SA_HACA2.5 and Gn_SA_HACA5, and to *Xc* = 490 nm in Gn_SA_HACA7.5, which can be explained by transformations in the crosslinked structure of the gelatin–alginate hydrogels due to the introduction of these humic acids. Due to the rearrangement of the three-dimensional network of hydrogels, the fluorescence quantum yields slightly decreased to Φ ≈ 33% for Gn_SA_HACA2.5 and to Φ ≈ 27% for Gn_SA_HACA5. However, as a result of the significant strengthening of the hydrogel network in Gn_SA_HACA7.5, a very significant increase in the fluorescence quantum yield to Φ ≈ 51% was observed. Unfortunately, a negative consequence of this strengthening is excessively high melting temperatures in the range of 55–60 °C, which makes calcium-containing hydrogels unsuitable for use in temperature-sensitive drug delivery.

**Figure 4:**
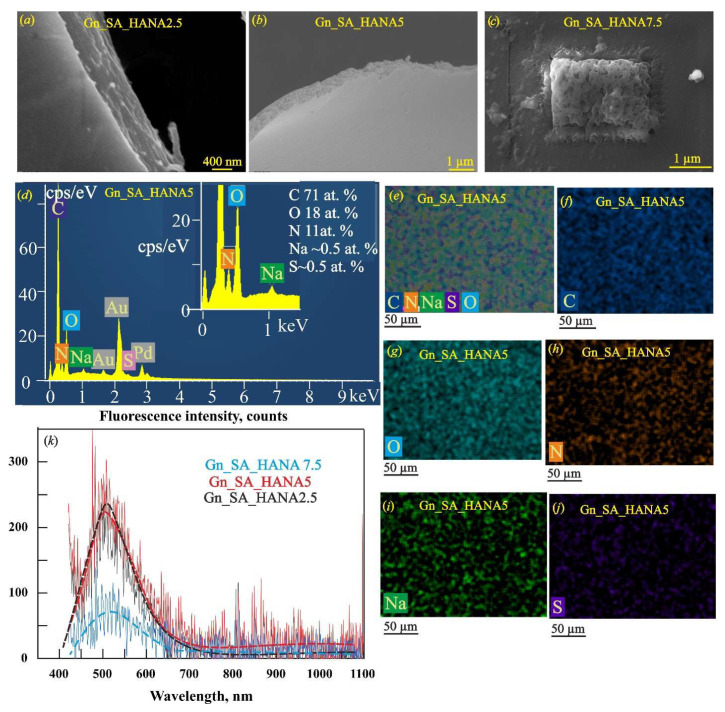
SEM images of dried gelatin–alginate hydrogels modified with HANA humic acids: (**a**) Gn_SA_HANA2.5; (**b**) Gn_SA_HANA5; (**c**) Gn_SA_HANA7.5. (**d**) EDS spectrum of the dried Gn_SA_HANA5 hydrogel. (**e**) Overall EDS map of the dried Gn_SA_HANA5 hydrogel. Elemental EDS mapping of individual elements in the dried Gn-SA-HANA5 hydrogel: (**f**) C; (**g**) O; (**h**) N; (**i**) Na; (**j**) S (all hydrogels are coated with 50 nm thick Au80Pd20 films). (**k**) Fluorescence emission spectra of dried gelatin–alginate hydrogels modified with HANA humic acids at an excitation wavelength of 405 nm.

Previously, based on rheological studies in [30], it was shown that HANA humic acids in the gelatin–alginate hydrogels Gn_SA_HANA2.5 and Gn_SA_HANA5 slightly increase the gel–sol transition temperature. The Gn_SA_HANA2.5 hydrogel has a *T_GS_* of ~36.9 °C, and the Gn_SA_HANA5 hydrogel has a *T_GS_* of ~37.2 °C. The above correlates well with the data [26] for gelatin hydrogels containing HANA, the mechanical and thermal properties of which were improved at a relatively low content of humic acids in GN hydrogels, up to a concentration of 13.33 (*w*/*w*)%, promoting the formation of tighter networks. X-ray diffraction analysis in [31] showed that in the dried Gn_SA_HANA2.5 hydrogel, the number of nanocrystalline segments increases compared to the dried Gn_SA hydrogel due to additional non-covalent HANA_Gn and HANA_SA bonds. Moreover, it was also shown in [30] that both Gn_SA_HANA2.5 and Gn_SA_HANA5 sols have lower kinematic viscosity at temperatures above *T_GS_* compared to the Gn_SA sol. According to [30], Gn_SA_HANA2.5 and Gn_SA_HANA5 hydrogels have lower swelling capacities compared to the Gn_SA hydrogel: the free swelling absorption capacity decreased from 8.3% for Gn_SA to 6% for Gn_SA_HANA2.5 and 7% for Gn_SA_HANA5. According to [26,27,30], all of the above may be the result of non-covalent interactions such as hydrogen bonds and hydrophobic and electrostatic interactions between the carbonyl groups of HANA and the hydroxyl groups of proline residues and the hydroxyproline and glycine amino groups of gelatin chains, which contribute to the formation of a denser gel network. As can be seen in [30,31,32], these gelatin–alginate hydrogels are brown in color because they contain large undissolved HANA particles up to tens of micrometers in size dispersed in the hydrogels due to the amphiphilic property of alginate. Figure 2b shows the reduced UV–visible light transmittance in the λ range of 250–500 nm for dried Gn_SA_HANA2.5, Gn_SA_HANA5, and Gn_SA_HANA7.5 hydrogel films with a thickness of ~0.5 mm, not exceeding 38%. This is confirmed by the photograph of the Gn_SA_HANA5 sample in Figure 2c. However, Figure 4 shows the top and cross-sectional SEM images, EDS spectrums, and EDS maps obtained for small areas (50 × 50 μm) of the gelatin–alginate hydrogels, where no undissolved HANA particles were observed. Figure 4a,b show the SEM images of the dry hydrogel washers Gn_SA_HANA2.5 and Gn_SA_HANA5, respectively, with smooth surfaces and layered cross-sections. It is evident that when recording using the SEM and EDS methods at an accelerating voltage of 10 kV, thin layers with a thickness of ~0.5 μm are peeled off from these samples under the action of an electron beam, which may be a consequence of the anisotropy of the morphology of the dried Gn_SA_HANA2.5 and Gn_SA_HANA5 hydrogels, namely their layered structure. It was demonstrated in [30] that high concentrations of HANA in the Gn_SA_HANA7.5 hydrogel increase the gel–sol transition temperature *T_G_**_S_*** to ~47.5 °C, i.e., much higher than the physiological temperature of 37 °C, which makes this hydrogel unsuitable for drug delivery. The change in the secondary structure of gelatin hydrogels containing high concentrations of HANA (more than 13.33 (*w*/*w*)%), which lost their triple-helix structures and showed an increase in random coil conformation, was described in [26]. This phenomenon was confirmed in [31] by X-ray diffractometric studies as a decrease in the number of nanocrystalline segments of polyguuronate units of alginate with a decrease in the average size of the nanocrystallites of polyguronate from 5.3 nm to 2.3 nm and an increase in HANA concentrations in the gelatin–alginate hydrogel from 2.5 wt.% to 7.5 wt.%. Accordingly, the dislocation density of polyguronate increased sharply from 0.04 lines/nm^2^ in the Gn_SA_HANA2.5 hydrogel to 0.19 lines/nm^2^ in the Gn_SA_HANA7.5 hydrogel [31]. Moreover, X-ray diffraction analysis in [31] showed the effect of HANA on gelatin nanocrystals and on the nanocrystalline polymannuronate component of alginate in the form of a decrease in the interplanar distances of gelatin and polymannuronate nanocrystals in the Gn_SA_HANA7.5 hydrogel, which confirms the occurrence of large tensile microstrains in small gelatin/polymannuronate nanocrystals ~0.7 nm in size. Accordingly, the high dislocation density in these small nanocrystals increased further to 2.78 lines/nm^2^, indicating the formation of a more disordered gel as a result of adding large amounts of HANA to the gelatin–alginate hydrogel. In addition, as the concentration of HANA humic acids in the gelatin–alginate hydrogel increased, X-ray diffraction showed an increase in amorphous halos due to the defective structure of the hydrogels. Finally, an additional X-ray diffraction peak was detected in the Gn_SA_HANA7.5 hydrogel, which corresponded to the small HANA nanocrystals with an average size of 1 nm [31]. As shown in [30], the free swelling capacity of the Gn_SA_HANA7.5 hydrogel increased to 13.6%. According to [26,27], this is due to the high affinity of HANA for water, which establishes preferential bonds with H_2_O molecules, preventing them from coordinating with gelatin chains and resulting in the secondary structure of gelatin losing its triple-helical structure and the gel network becoming weaker and larger, as illustrated in [12] for gelatin-based drug delivery systems.

Another result of the high swelling property of HANA is the retention of water molecules even in the naturally dried Gn_SA_HANA7.5 hydrogel, which is shown in the SEM image of Figure 4c as the swelling and “boiling” of this hydrogel under the electron beam at an accelerating voltage of 10 kV during SEM and EDS measurements. Thus, only Gn_SA_HANA2.5 and Gn_SA_HANA5 were selected in [30] as suitable thermo-responsive gelatin–alginate hydrogels containing HANA for drug delivery. The smooth, even, and uniform surface, with a fairly uniform element distribution, is demonstrated by the overall EDS map of the dried Gn_SA_HANA5 hydrogel in Figure 4e and its elemental EDS maps of carbon, oxygen, nitrogen, sodium, and sulfur in Figure 4f–j, respectively. The fluorescence emission spectra of dried gelatin–alginate hydrogels modified with HANA humic acids in Figure 4k and the data in Table 2 for the dried Gn_SA_HANA2.5, Gn_SA_HANA5, and Gn_SA_HANA7.5 hydrogels revealed the quenching of autofluorescence, which, in accordance with [19,25], is a signal of a significant change in the internal structure of the gel upon modification by HANA. In the series of Gn_SA, Gn_SA_HANA2.5, Gn_SA_HANA5, and Gn_SA_HANA7.5, the intensity of the fluorescence peak decreased monotonically from ~1120 to ~235, ~220, and ~70 counts, respectively. Accordingly, the fluorescence quantum yields of the dried hydrogels decreased to Φ ≈ 12% for Gn_SA_HANA2.5, Φ ≈ 10% for Gn_SA_HANA5, and Φ ≈ 3% for Gn_SA_HANA7.5. In addition, the center of the fluorescence peak *Xc* was red-shifted from 500 nm in Gn-SA to 517 nm in Gn_SA_HANA2.5, 518 nm in Gn_SA_HANA5, and 524 nm in Gn_SA_HANA7.5, respectively. In [19,25], it was indicated that the quenching of hydrogel autofluorescence may be a consequence of the formation of a strong complex between the functional groups of the hydrogel and some specific metal ions. On the other hand, according to [19], the autofluorescence intensity in carbonyl-containing gels shows a close correlation with the water content, since the fluorescence intensity increases with decreasing water content in the gels, realizing the emission caused by aggregation in a water-deficient environment. Thus, the hydrophilicity of HANA and the ability of these humic acids to retain water in the dried gel, demonstrated in this work by the SEM results in Figure 4c, are the most obvious reasons for the quenching of autofluorescence in HANA-containing gelatin–alginate hydrogels.

**Figure 5:**
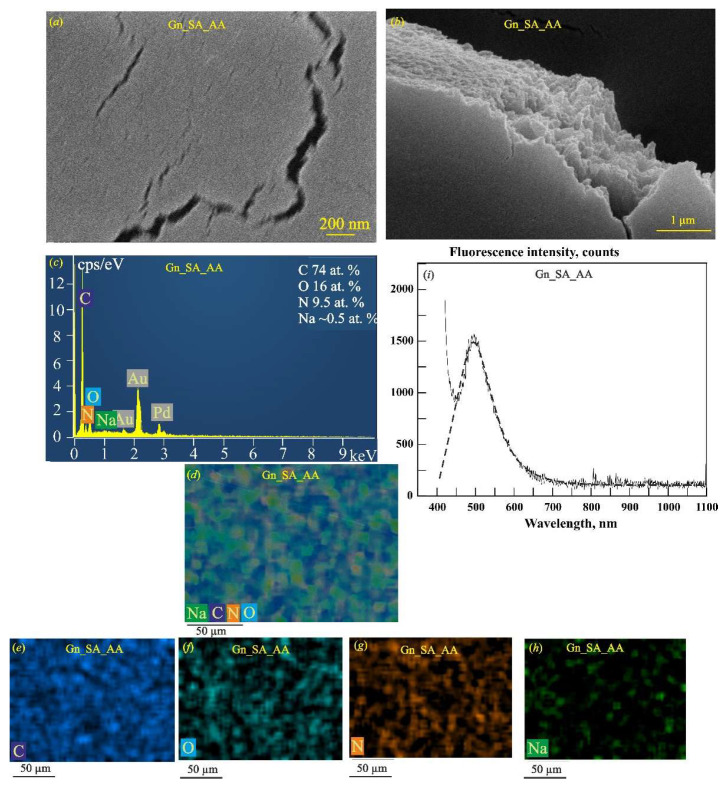
SEM images of a dried gelatin–alginate hydrogel loaded with aminocaproic acid (Gn_SA_AA): (**a**) top view and (**b**) cross-section. (**c**) EDS spectrum of the dried Gn_SA_AA hydrogel. (**d**) Overall EDS map of the dried Gn_SA_AA hydrogel. Elemental EDS mapping of individual elements in the dried Gn_SA_AA hydrogel: (**e**) C; (**f**) O; (**g**) N; (**h**) Na (the hydrogel is coated with a 50 nm thick Au80Pd20 film). (**i**) Fluorescence emission spectrum of the dried gelatin–alginate hydrogel Gn-SA-AA at an excitation wavelength of 405 nm.

As shown earlier [32], loading thermosensitive gelatin–alginate hydrogels with the hemostatic drug aminocaproic acid does not significantly affect the rheological properties. According to the data [30,32], the kinematic viscosity of the hydrogel near the gel–sol transition temperature increased with the addition of AA powder by 20% by weight from ~500 mm^2^/s for Gn_SA to ~600 mm^2^/s for Gn_SA_AA. In [32], it was also shown that the gel–sol transition temperature in the Gn_SA_AA hydrogel is equal to the physiological temperature of 37 °C. Figure 5a,b show the top-view and cross-sectional SEM images of the dried Gn_SA_AA hydrogel, respectively. Similar to the dried Gn_SA hydrogel in Figure 1b, the dried Gn_SA_AA hydrogel in Figure 5a has large cracks that are attributed to the strong compression of the washer during its natural drying process, which may be the cause of the unwanted wound contracture and contour deformation when the hydrogel dressing is used for wound healing. The cross-section of Gn_SA_AA in Figure 5b confirms its layered morphology. As can be seen from the EDS spectrum in Figure 5c, the composition of Gn_SA_AA includes carbon and oxygen from Gn, SA, and AA, nitrogen from Gn and AA, and sodium from SA. The overall EDS map in Figure 5d and the corresponding elemental EDS maps in Figure 5e–h show a fairly uniform distribution of C, O, N, and Na throughout the sample. The autofluorescence spectrum of the dried aminocaproic acid-impregnated gelatin–alginate hydrogel Gn_SA_AA in Figure 5i has a broad peak at *Xc* = 498 nm, i.e., near *Xc* = 500 nm for the dried Gn_SA hydrogel, which, due to the higher kinematic viscosity of the Gn_SA_AA hydrogel, was more intense compared to the corresponding emission of the dried Gn_SA hydrogel (1500 counts versus 1120 counts). Accordingly, the fluorescence quantum yield of the dried Gn_SA_AA hydrogel increased to Φ ≈ 43%. The optical transmittance spectrum in the λ range of 250–500 nm of the dried Gn_SA_AA hydrogel film with a thickness of ~0.5 mm in Figure 2d demonstrates its reduced UV–visible light transmittance of up to 60% compared to *T* ≈ 90% for Gn_SA, which is explained by light scattering due to the high content of aminocaproic acid.

**Figure 6:**
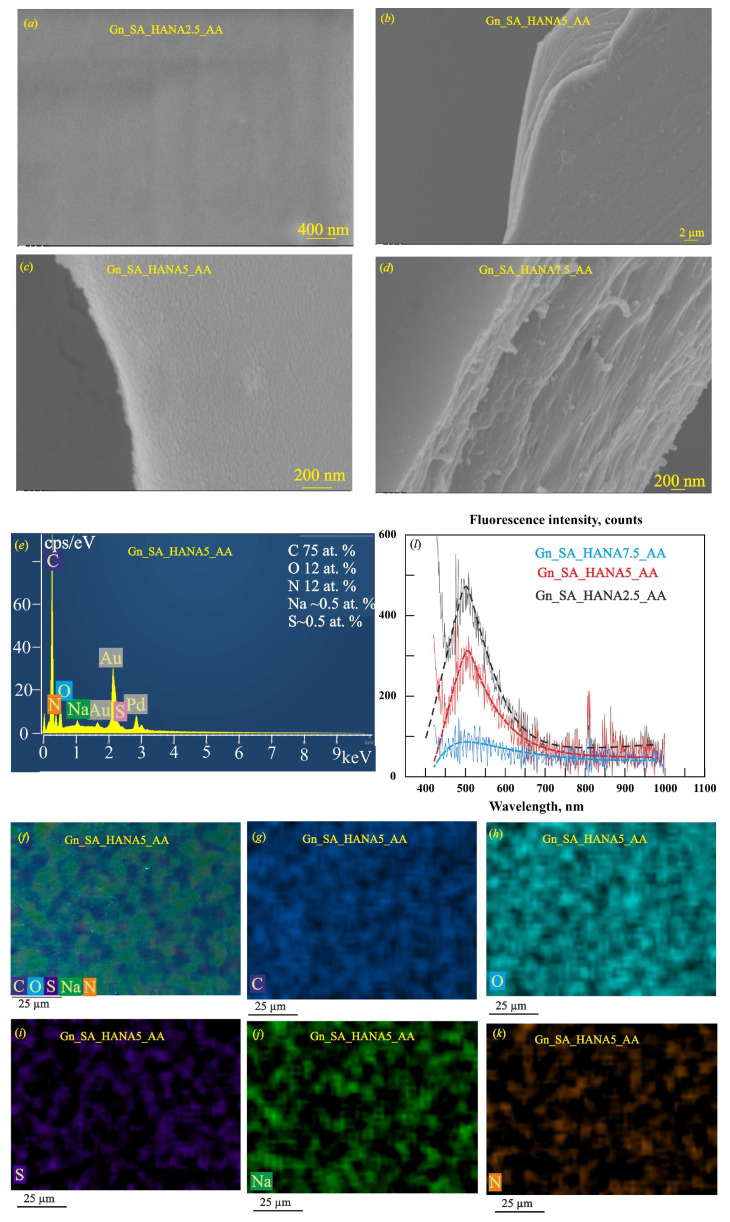
SEM images of dried gelatin–alginate hydrogels modified with HANA humic acids and loaded with aminocaproic acid: (**a**) Gn_SA_HANA2.5_AA; (**b**,**c**) Gn_SA_HANA5_AA; (**d**) Gn_SA_HANA7.5_AA. (**e**) EDS spectrum of the dried Gn_SA_HANA5_AA hydrogel. (**f**) Overall EDS map of the dried Gn_SA_HANA5_AA hydrogel. Elemental EDS mapping of individual elements in the dried Gn_SA_HANA5_AA hydrogel: (**g**) C; (**h**) O; (**i**) S; (**j**) Na; (**k**) N (all hydrogels are coated with 50 nm thick Au80Pd20 films). (**k**) Fluorescence emission spectra of dried gelatin–alginate hydrogels modified with HANA humic acids and loaded with AA at an excitation wavelength of 405 nm.

Figure 6a–d show the SEM images of dried gelatin–alginate hydrogels loaded with the hemostatic drug aminocaproic acid and modified with HANA humic acids. They exhibit smooth and even surfaces, layered cross-sections, and the anisotropic morphology of dried Gn_SA_HANA2.5-AA, Gn_SA_HANA5_AA, and Gn_SA_HANA7.5_AA hydrogels, whose ~2 μm thick layers were exfoliated during SEM and EDS observations. Since all these systems had a layered and homogeneous morphology, this indicated a well-dispersed AA filler due to the high solubility of aminocaproic acid in the corresponding sols. Comparison of data [30,32] showed that after adding AA powder in an amount of 20% by weight, the kinematic viscosity of the hydrogels near the gel–sol transition temperature increased from ~550 mm^2^/s for Gn_SA_HANA2.5 and Gn_SA_HANA5 to ~650 mm^2^/s for Gn_SA_HANA2.5_AA and to ~700 mm^2^/s for Gn_SA_HANA5_AA. In [32], it was shown that the gel–sol transition temperatures of the Gn_SA_AA, Gn_SA_HANA2.5_AA, and Gn_SA_HANA5_AA hydrogels were equal to the physiological temperature of 37 °C, which allowed the hydrogel to soften and melt on human skin or inside the wound, ensuring the delivery of the hemostatic drug AA in accordance with [31].

As can be seen from the SEM images in Figure 6a–c, due to the crosslinked structure with appropriate strength and increased viscosity, the Gn_SA_HANA2.5_AA and Gn_SA_HANA5_AA hydrogels do not crack because they have a small contracture during natural drying. Experiments in [32] showed that by increasing the content of HANA humic acids to 7.5 wt.%, a thermosensitive gelatin–alginate hydrogel with aminocaproic acid Gn_SA_HANA7.5_AA was obtained, the *T_G_**_S_*** of which increased to ~47.5 °C. As can be seen in Figure 6d, due to the increased viscosity of the Gn_SA_HANA7.5_AA sol, neither cracking nor swelling and “boiling” were observed for the naturally dried hydrogel under an electron beam at an accelerating voltage of 10 kV in SEM and EDS measurements. The EDS spectrum of the dried Gn_SA_HANA5_AA hydrogel in Figure 6e shows that it contains carbon and oxygen from Gn, SA, HANA, and AA, nitrogen from Gn and AA, sulfur from HANA, and sodium from SA and HANA. The overall EDS map in Figure 6f and the corresponding elemental EDS maps in Figure 6g–j show a fairly uniform distribution of C, O, S, Na, and N throughout the sample. The optical transmission spectra of dried gelatin–alginate hydrogel films of thickness ~0.5 mm containing HANA and AA in Figure 2d demonstrate a decrease in their transparency for UV–visible light with an increase in HANA content from 2.5 wt.% to 7.5 wt.%, which is in good agreement with the brown color of the films visible in the photograph of the Gn_SA_HANA2.5_AA sample in Figure 2c. In accordance with the increased kinematic viscosity of the Gn_SA_HANA2.5_AA, Gn_SA_HANA5_AA, and Gn_SA_HANA7.5_AA sols, the fluorescence emission spectra of their dried hydrogels in Figure 6k and the data in Table 2 revealed a slight decrease in HANA-induced autofluorescence quenching. However, in the series of Gn_SA_AA, Gn_SA_HANA2.5_AA, Gn_SA_HANA5_AA, and Gn_SA_HANA7.5_AA, the fluorescence peak intensity decreased monotonically from ~1500 to ~480, ~320, and ~75 counts, respectively. The fluorescence quantum yields are Φ ≈ 10% for Gn_SA_HANA2.5_AA, Φ ≈ 8% for Gn_SA_HANA5_AA, and Φ ≈ 5% for Gn_SA_HANA7.5_AA. The fluorescence peak center *Xc* is slightly red-shifted with increasing HANA content from 498 nm in Gn_SA_AA to 500 nm in Gn_SA_HANA2.5_AA, 510 nm in Gn_SA_HANA5_AA, and 517 nm in Gn_SA_HANA7.5_AA, respectively.

## 3. Conclusions

This paper presents thermosensitive, hemostatic, and wound-healing biopolymer hydrogels that have the ability to autofluorescence due to internal fluorophores of alginate and gelatin. The crosslinked dried gelatin–alginate hydrogel Gn_SA demonstrated a fluorescence quantum yield of up to 35%. The disadvantage of this hydrogel is its large contracture upon drying, which was shown in SEM images as large cracks. Modification of this hydrogel with HACA humic acids containing calcium ions to improve its anti-inflammatory activity led to additional shrinkage of the hydrogel network, which was displayed in SEM images as many small cracks due to contracture. In dried hydrogels of the Gn_SA_HACA family, due to coagulation caused by calcium ions, a decrease in transparency in the near-ultraviolet and visible regions of the spectrum and a significant increase in the intensity of autofluorescence with an increase in its quantum yield to 51% at a content of 7.5 wt.% HACA were found. However, since the gel–sol transition temperature of Gn_SA_HACA-family hydrogels is much higher than the physiological temperature, they cannot be used to deliver hemostatic drugs to a wound. Therefore, for this purpose, HANA humic acids were used in hydrogels, which also have wound-healing properties but contain sodium ions instead of calcium ions. The effect of these humic acids in gelatin–alginate hydrogels in the form of the rearrangement of three-dimensional gel networks is manifested in fluorescence quenching and a decrease in the fluorescence quantum yield to 3–12% in dried Gn_SA_HANA-family hydrogels. At the same time, the excess HANA content in the Gn_SA_NANA7.5 hydrogel is manifested in SEM images as swelling and “boiling”, which indicates water retention by hydrophilic HANA humic acids. The optimal concentrations of HANA in gelatin–alginate hydrogels were 2.5 wt.% and 5 wt.%, which provide a gel–sol transition temperature of about 37 °C. The absence of fluorescence quenching upon introduction of the hemostatic drug aminocaproic acid into gelatin–alginate hydrogels indicates that AA does not destroy the three-dimensional networks formed by molecules of gelatin, alginate, and HANA humic acids, the gel–sol transition temperature for which remained at the physiological level. The fluorescence quantum yield of the dried gelatin–alginate hydrogel loaded with 20 wt.% aminocaproic acid was as high as ~43%. Evidence of the minor contracture of Gn_SA_HANA2.5_AA and Gn_SA_HANA5_AA hydrogels is the preservation of a smooth, even surface without cracks and a layered structure in SEM images. The above dried gelatin–alginate hydrogels containing HANA and 20 wt.% aminocaproic acid have a fluorescence quantum yield of 8–10%. Thus, the new approach of using fluorescence emission spectra, along with the analysis of morphological features, optical properties, and elemental composition of the dried hydrogel used in this work, turned out to be an additional tool for monitoring the ability of hydrogels for the thermosensitive delivery of a hemostatic drug and subsequent wound healing without major contracture of the wound dressing. In addition, the described new intelligent property of gelatin–alginate hydrogels with the modifiers and fillers presented here, such as autofluorescence, opens up the prospect of the wider use of biocompatible and biodegradable intelligent hydrogel materials based on gelatin–alginate in the designs of soft robots and temperature and light sensors, for which biopolymer hydrogels can act as active primary materials with the additional advantages of transparency and antimicrobial activity. Our next work will be devoted to the development of intelligent, biopolymer hydrogel transdermal microneedle patches based on gelatin and sodium alginate modified with HANA and impregnated with aminocaproic acid, obtained by sol–gel technology using a 3D-printed mold template similar to those presented in our recent work [39]. This will improve the penetration of drugs into the wound. Biopolymer hydrogel transdermal patches made of Gn_SA_HANA2.5_AA and Gn_SA_HANA5_AA with conical microneedles and with microneedles in the form of biomimetic adhesive protrusions will increase adhesion to human skin and ensure the successful delivery of drugs.

## 4. Materials and Methods

In this work, we used biopolymer edible gelatin, brand R-11 (TM Mriya, PJSC Ukroptbakalia, Chernihiv, Ukraine), and sodium alginate (C_6_H_7_O_6_Na)_n_ (Lianyungang Fengyun Seaweed Manufacturer Co., Ltd., Ganyu, China). HANA and HACA humic acids were obtained by extraction from brown coal with a solution of sodium pyrophosphate Na_4_P_2_O_7_ (Haihang Industry Co., Ltd., Jinan, China) and further extraction with sodium hydroxide (Shandong Luxian Chemical Products Co., Ltd., Yantai, China)—1 wt.% NaOH for HANA and calcium hydroxide (Guangxi Huitong New Materials Co., Ltd., Guilin, China) 0.2 wt.% Ca(OH)_2_ for HACA—followed by precipitation with hydrochloric acid HCl (Qingdao Hisea Chem Co., Ltd., Qingdao, Shandong, China) according to our own method described in [30]. Aminocaproic acid C_6_H_13_NO_2_ is produced by UmanHimTrade Co., Uman, Ukraine.

When preparing biopolymer hydrogels, the calculated amount of Gn was placed in distilled water preheated to 90 ± 2 °C and stirred in a water bath with a VEVOR 85-2 magnetic stirrer with heating in order to obtain a pure Gn sol. Then, SA was added to the Gn sol and stirred with this magnetic stirrer until a homogeneous Gn-SA sol was obtained. Before adding to the Gn-SA sol, HANA and HACA humic acids were partially dissolved in aqueous alkaline solutions of 1 wt.% NaOH and 0.2 wt.% Ca(OH)_2_, respectively. To load the hydrogels with aminocaproic acid, 10.18 g of AA powder was added to 50 mL of the corresponding sol sample, heated to a temperature above 50 °C, and thoroughly mixed until a suspension was obtained.

Then, 1.8 g of each hydrogel was naturally dried in a special mold, as shown in [30], for 48 h to obtain dry hydrogel washers with a diameter of 1.5 cm and a height of about 0.1 cm for their subsequent characterization. In addition, ~0.5 mm thick hydrogel films were obtained on glass substrates using the drop-casting method and following natural drying.

The morphology and chemical composition of the dry hydrogel washers were studied by scanning electron microscopy (SEM) and energy-dispersive X-ray spectrometry (EDS) using a Zeiss ULTRA Plus SEM (ZEISS, Jena, Germany) with a secondary electron (SE) detector equipped with an OXFORD X-Max 20 EDS detector (Oxford Instruments, Abingdon, UK), providing elemental mapping over areas of about 50 × 50 μm. Taking into account the dielectric properties of biopolymer hydrogels, before their analysis using SEM and EDS, 50 nm thick Au80Pd20 alloy films were applied to the surface of the hydrogels using the RF magnetron sputtering method. SEM images and EDS spectra were recorded at an accelerating voltage of 10 kV. The EDS maps of the samples were superpositions of the signal obtained from the electron backscatter detector (colored white) and the intensities of the characteristic lines of the elements C, O, N, Na, and Ca (colored in shades of the corresponding color). The resolution of the EDS map was 500 × 500 pixels.

The optical transmittance spectra T(λ) of hydrogel films on glass substrates were recorded relative to bare glass substrates in the wavelength range λ from 250 to 500 nm using a LAMBDA 35 PerkinElmer spectrophotometer (PerkinElmer Life and Analytical Sciences, Shelton, CT, USA).

To analyze the autofluorescence of biopolymer hydrogels, the fluorescence emission spectra of dry hydrogel washers were excited by laser radiation with a wavelength of 405 nm at room temperature and recorded using a BLACK-Comet CXR-SR-25 fiber-optic spectrometer. The fluorescence quantum yield (Φ) was defined as the percentage ratio of the number of emitted photons to the number of absorbed photons based on the integrated intensity of the emitted light to the integrated intensity of the excited light.

## Figures and Tables

**Table 1:** Biopolymer hydrogels studied in this work.

Hydrogel Symbol	Hydrogel Composition, wt.%				
	Gn	SA	HANA	HACA	AA
Gn_SA	14	6.4	-	-	-
Gn_SA_HANA2.5	14	6.4	2.5	-	-
Gn_SA_HANA5	14	6.4	5	-	-
Gn_SA_HANA7.5	14	6.4	7.5	-	-
Gn_SA_HACA2.5	14	6.4	-	2.5	-
Gn_SA_HACA5	14	6.4	-	5	-
Gn_SA_HACA7.5	14	6.4	-	7.5	-
Gn_SA_AA	14	6.4		-	20
Gn_SA_HANA2.5_AA	14	6.4	2.5	-	20
Gn_SA_HANA5_AA	14	6.4	5	-	20
Gn_SA_HANA7.5_AA	14	6.4	7.5	-	20

**Table 2:** Autofluorescent and thermosensitive properties of biopolymer hydrogels.

Hydrogel	Fluorescence Data of Dry Hydrogels				Melting Point of Hydrogel *T_GS_*, °C [30,32]
	Maximum Fluorescence Peak Intensity, Counts	Gaussian Fitting Using Origin Pro 8		Fluorescence Quantum Yield, Φ, %	
		Peak Center *Xc*, nm	FWHM, nm		
Gn_SA	1120 ± 5	500 ± 2	124 ± 2	35 ± 3	36.4 ± 0.4
Gn_SA_HACA2.5	1900 ± 5	496 ± 2	127 ± 2	33 ± 3	55.0 ± 0.3
Gn_SA_HACA5	1800 ± 5	496 ± 2	126 ± 2	27 ± 3	58.0 ± 0.2
Gn_SA_HACA7.5	2300 ± 5	490 ± 2	140 ± 2	51 ± 3	60.0 ± 0.4
Gn_SA_HANA2.5	235 ± 5	517 ± 2	137 ± 2	12 ± 3	36.9 ± 0.4
Gn_SA_HANA5	220 ± 5	518 ± 2	143 ± 2	10 ± 3	37.2 ± 0.4
Gn_SA_HANA7.5	70 ± 5	524 ± 2	156 ± 2	3 ± 3	47.5 ± 0.4
Gn_SA_AA	1500 ± 5	498 ± 2	122 ± 2	43 ± 3	37.0 ± 0.4
Gn_SA_HANA2.5_AA	480 ± 5	500 ± 2	154 ± 2	10 ± 3	37.0 ± 0.4
Gn_SA_HANA5_AA	320 ± 5	510 ± 2	145 ± 2	8 ± 3	37.0 ± 0.3
Gn_SA_HANA7.5_AA	75 ± 5	517 ± 2	145 ± 2	5 ± 3	47.0 ± 0.4

## Data Availability

The data are available from the author for correspondence upon reasonable request.
